# Insights into IAV Replication and Lipid Metabolism in Suspension-Adapted MDCK-STAT1-KO Cells

**DOI:** 10.3390/vaccines13020106

**Published:** 2025-01-22

**Authors:** Qian Ye, Hong Yao, Zhiying Xiao, Liang Zhao, Wen-Song Tan

**Affiliations:** 1 State Key Laboratory of Bioreactor Engineering, East China University of Science and Technology, Shanghai 200237, China; qy@mail.ecust.edu.cn (Q.Y.);; 2 Shanghai Collaborative Innovation Center for Biomanufacturing Technology (SCIBT), Shanghai 200237, China; 3 Shanghai BioEngine Sci-Tech Co., Ltd., Shanghai 201203, China

**Keywords:** influenza vaccine, STAT1 gene knockout, suspension MDCK cells, host immune responses, lipid metabolism

## Abstract

Objectives: The industrial production of influenza vaccines is facing significant challenges, particularly in improving virus production efficiency. Despite advances in cell culture technologies, our understanding of the production characteristics of high-yield suspension cell lines remains limited, thereby impeding the development of efficient vaccine production platforms. This study aims to investigate the key features of STAT1 knockout suspension-adapted MDCK cells (susMDCK-STAT1-KO) in enhancing influenza A virus (IAV) production. Methods: Suspension-adapted susMDCK-STAT1-KO cells were compared to suspension-adapted wild-type MDCK cells (susMDCK) for IAV production. Virus quantification, gene expression analysis, and cholesterol deprivation assays were performed. Metabolite profiles, viral RNA quantification, and lipid and dry weight measurements were also conducted to assess the viral replication and release efficiency. Results: The susMDCK-STAT1-KO cells exhibited significantly improved virus adsorption (64%) and entry efficiency (75%) for the H1N1 virus, as well as accelerated viral transcription and replication for both the H1N1 and H9N2 viruses. Virus release was identified as a limiting factor, with a 100-fold higher intracellular-to-extracellular viral RNA ratio. However, the STAT1-KO cells showed a 2.39-fold higher release rate (750 virions/cell/h) and 3.26-fold greater RNA release for the H1N1 virus compared to wild-type cells. A gene expression analysis revealed enhanced lipid metabolism, particularly cholesterol synthesis, as a key factor in viral replication and release. Cholesterol deprivation resulted in reduced viral titers, confirming the critical role of intracellular cholesterol in IAV production. Conclusions: This study demonstrates the enhanced influenza virus production capacity of susMDCK-STAT1-KO cells, with significant improvements in viral yield, replication, and release efficiency. The findings highlight the importance of STAT1-mediated immune modulation and cholesterol metabolism in optimizing virus production. These insights provide a foundation for the development of more efficient vaccine production platforms, with implications for large-scale industrial applications.

## 1. Introduction

Influenza outbreaks pose a significant threat to global health, with vaccination recognized as the most cost-effective preventive strategy [1]. The Mardin–Darby canine kidney (MDCK) cell line serves as a key platform for influenza virus vaccine production [2]. Advances in understanding viral replication and cellular stress have enabled the development of genetically engineered MDCK cell lines, targeting specific pathways to enhance virus production [3]. Among these, the JAK/STAT signaling pathway is critical for host antiviral responses, driving the expression of numerous interferon-stimulated genes (ISGs) [4]. The MDCK-STAT1-KO cells, generated through CRISPR/Cas9-mediated STAT1 gene knockout, demonstrated significantly increased virus titers [5]. However, MDCK-STAT1-KO cells are currently limited by their adherent culture mode, restricting their scalability for industrial applications [6]. Transitioning to a single-cell suspension culture is essential; yet, the physiological and growth characteristics of MDCK-STAT1-KO cells under suspension conditions remain unexplored. Moreover, suspension adaptation may alter the cell surface receptor levels and virus production characteristics [7,8,9], raising questions about whether these changes affect the virus yield of MDCK-STAT1-KO cells.

To address the challenges in influenza vaccine production, this study evaluates the performance of a suspension-adapted MDCK-STAT1 knockout (susMDCK-STAT1-KO) cell line compared to suspension-adapted wild-type MDCK (susMDCK) cells. The findings demonstrate that susMDCK-STAT1-KO cells exhibit markedly superior H1N1 virus adsorption (64%) and entry efficiency (75%), alongside accelerated viral transcription, replication, and earlier release for both H1N1 and H9N2 viruses. Despite identifying virus release as a production bottleneck, evidenced by an intracellular-to-extracellular viral RNA ratio of nearly 100-fold higher, the STAT1-KO cells achieve a H1N1 virus release rate of 750 virions per cell per hour and an RNA release ratio of 2.8%, representing 2.39-fold and 3.26-fold increases, respectively, over susMDCK cells. Further analysis reveals that attenuated innate immune responses in susMDCK-STAT1-KO cells facilitate enhanced viral replication, while upregulated lipid metabolism—particularly cholesterol synthesis—drives rapid viral replication and efficient release. Cholesterol is highlighted as a critical factor supporting influenza virus production.

Overall, these results deepen our understanding of virus production mechanisms in suspension MDCK cells, providing a strong foundation for optimizing cell engineering strategies and improving vaccine manufacturing processes. This work underscores the industrial potential of susMDCK-STAT1-KO cells as a robust platform for influenza vaccine production.

## 2. Materials and Methods

### 2.1. Medium and Cell Line

Adherent MDCK cells (ATCC and CCL-34) and adherent MDCK-STAT1-KO cells (ATCC and CCL-34-VHG™) were cultured in T-flasks using DMEM (BioEngine, Shanghai, China) supplemented with 10% (*v*/*v*) fetal bovine serum (Biosun, Shanghai, China) at 37 °C in a humidified incubator containing 5% CO_2_, for the 50% tissue culture infectious dose (TCID_50_) assay. The adherent MDCK cells were adapted to suspension culture by the serum reduction and serial passaging approach in serum-free SF003 medium (BioEngine, Shanghai, China) as previously reported [10], and the resulting susMDCK cells and susMDCK-STAT1-KO cells were cultured in SF003 medium.

### 2.2. Virus Quantification

Avian influenza A virus (A/chicken/Guangxi/SIC6/2013(H9N2)) and human influenza A virus (A/human/PR8(H1N1)) were generously provided by Guangdong Wens Dahua’nong Biotechnology Co., LTD (Zhaoqing, China) and Shanghai Biology Institute, respectively (Shanghai, China).

Hemagglutination (HA) assays were performed following established protocols [11]. Total virus particle concentrations were estimated based on the assumption that hemagglutination occurs when the number of virus particles equals the number of erythrocytes [12]. The endpoint dilution showing complete hemagglutination was recorded as the titer, and HA assay titers were expressed as log2 HA units per 100 μL (log2 HAU/100 μL). The calculations incorporated HA titer values and erythrocyte concentrations of 2 × 10^7^ cells/mL

The TCID_50_ assay was conducted as described previously [13]. Instead of directly observing cytopathic effects (CPEs), HA assays were employed to identify virus-positive wells. Infectious virion counts per mL were calculated using the Reed–Muench method [14]. The TCID_50_ titer of the seed virus used for infection was determined on adherent MDCK cells.

### 2.3. Virus Infection

For synchronized single-cycle infections, suspension MDCK cells were batch-cultured until a viable cell density of (10–12) × 10^6^ cells/mL was achieved. The cells were centrifuged at 650× *g* for 5 min, and the supernatant was discarded. The resulting cell pellet was resuspended in fresh medium to a viable cell density of (4–6) × 10^6^ cells/mL. Virus infection experiments were carried out in 125 mL shake flasks with a working volume of 30 mL. The virus suspension was added at a multiplicity of infection (MOI) of 10, along with N-tosyl-L-phenylalanine chloromethyl ketone (TPCK)-treated trypsin at a concentration of 5 μg/mL. The flasks were incubated at 37 °C, 5% CO_2_, and 130 rpm for 1 h. Following incubation, cells were centrifuged, and the supernatant was removed. The cell pellet was washed twice with PBS, resuspended in fresh medium without TPCK-treated trypsin, and incubated under the same conditions.

The efficiency of virus attachment and entry was assessed during synchronized infection. Samples were collected at 0 h and 1 h post-infection (hpi) to measure HA titers. The change in viral particle count in the supernatant between 0 and 1 hpi was used to calculate total viral entry efficiency within the first hour of infection. For attachment control, cells were pre-treated with 0.1% sodium azide to inhibit viral internalization [15]. This approach allowed assessment of viral attachment efficiency while preventing entry into cells. Additionally, a negative control was included by exposing virus-free culture medium to the same conditions to monitor potential degradation of viral particles.

### 2.4. Cholesterol Deprivation Assay

To reduce cellular cholesterol levels, methyl-β-cyclodextrin (MβCD) was added to SF003 medium at final concentrations of 0, 5, 10, and 15 mg/mL. Suspension MDCK cells were cultured in the prepared media for 12 h, after which samples were collected to measure intracellular cholesterol content.

Following treatment, cells from each experimental group were centrifuged at 650× *g* for 5 min, and the cell pellets were resuspended in fresh medium. Subsequently, cells were infected with H9N2 virus at a MOI of 1. After 6 h of infection, the HA titers of each experimental group were determined.

### 2.5. Cell Counting

The viable cell density, viability, and cell size were evaluated using CounterStar cell analyzer (Countstar, Shanghai, China). The membrane area was calculated according to the measured cell size and the number of viruses the cell released, assuming they were spherical (100 nm).

### 2.6. Measurement of Metabolites

Extracellular metabolites regarding glucose and lactate were measured by corresponding kits (Nanjing Jiancheng Bioengineering Institute, Nanjing, China) following their instructions. Extracellular amino acids were measured using an Agilent AdvanceBio AAA C18 column based on Agilent’s diode array detector (DAD), for ortho-phthalaldehyde/9-fluorenyl-methyl chloroformate (OPA/FMOC) derivatization reagents. Before the sample detection, its concentration was diluted to the linear range of amino acid detection, and the protein in the sample should be precipitated with 10% (*w*/*v*) trichloroacetic acid solution. The High-Performance Liquid Chromatography procedures are detailed in Agilent’s amino acid analysis instructions. The cellular and supernatant cholesterol was measured by the Amplex Red Cholesterol and Cholesteryl Ester Assay Kit (Beyotime, Shanghai, China).

### 2.7. Determination of Cell Dry Weight and Lipid Dry Weight

For cell dry weight measurement, suspension cells (1 × 10^8^) were collected, washed, and vacuum-dried at 40 °C until a constant weight was achieved.

For lipid dry weight measurement, suspension cells (1 × 10^8^) were collected, washed, and extracted using a 1:20:35 mixture of ultrapure water, methanol, and chloroform. The extract was filtered with 0.8 mm filter paper, and the filtrate and subsequent methanol-chloroform washes were combined. Phase separation was induced with saline, and the upper phase was discarded. This step was repeated twice with additional chloroform, methanol, and NaCl solution. The lower phase was vacuum-dried at 40 °C until a constant weight was reached.

### 2.8. Quantitative PCR

RNAs were extracted using TRNzol Universal (Tiangen, Beijing, China) and reverse-transcribed into cDNA using the FastKing gDNA Dispelling RT SuperMix (Tiangen, Beijing, China). Quantitative PCR (qPCR) was performed with Hieff UNICON^®^ Universal Blue qPCR SYBR Green Master Mix (Yeasen, Shanghai, China). The mRNA expressions of GAPDH, IFN-β, IFITM3, SOCS3, Mx1, OAS1, IRF9, RSAD2, and BST2 were quantified by standard qPCR protocol. The vRNA, cRNA, and mRNA expressions of the influenza virus were quantified using strand-specific real-time RT-PCR methods as described previously [16]. Transcript expression levels were normalized to glyceraldehyde-3-phosphate dehydrogenase (GAPDH) expression. Primer sequences used in the assays are provided in Table A1 and Table A2.

### 2.9. Statistical Analysis

The statistical significance was assessed using the Student’s *t*-test, and *p*-value of <0.05 was considered statistically significant, with * indicating 0.01 < *p* < 0.05, ** indicating 0.001 < *p* < 0.01, and *** indicating *p* < 0.001.

## 3. Results and Discussion

### 3.1. Enhanced Virus Productivity in STAT1 Knockout MDCK Cells

**Figure 1:**
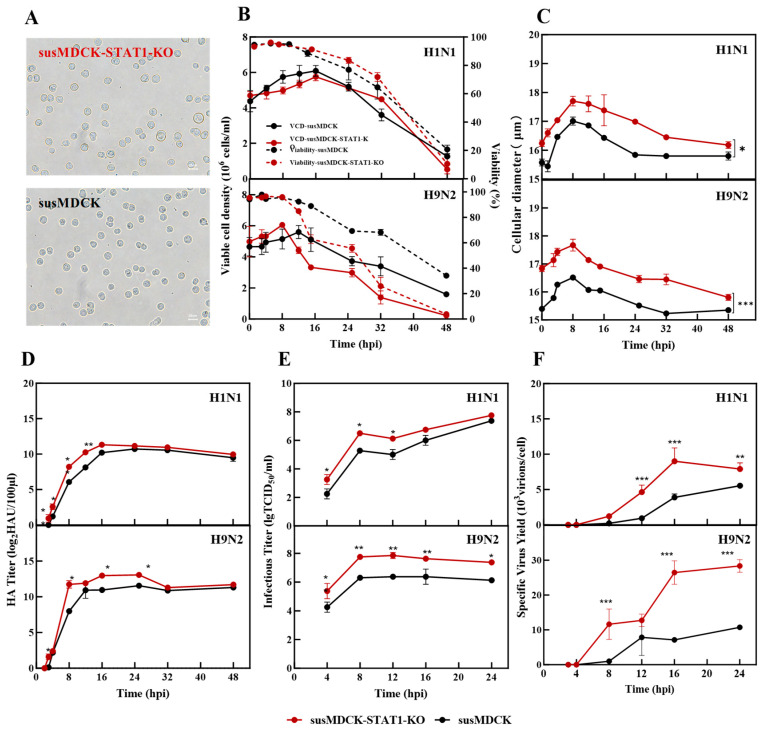
Morphology and IAV production of susMDCK-STAT1-KO and susMDCK cells: (**A**) morphological characteristics of cells under normal growth conditions; (**B**) changes in viable cell density and cell viability after synchronized infection with IAV; (**C**) changes in cell diameter following synchronized infection with IAV; (**D**) HA titers of viral supernatants harvested at different time points post-synchronized IAV infection; (**E**) infectious titers of viral supernatants harvested at various time points post-synchronized IAV infection; and (**F**) changes in specific virus yield (SVY) of viral supernatants collected at different time points post-synchronized IAV infection. Synchronized infections were performed with an MOI of 10, with * indicating 0.01 < *p* < 0.05, ** indicating 0.001 < *p* < 0.01, and *** indicating *p* < 0.001.

**Figure A1:**
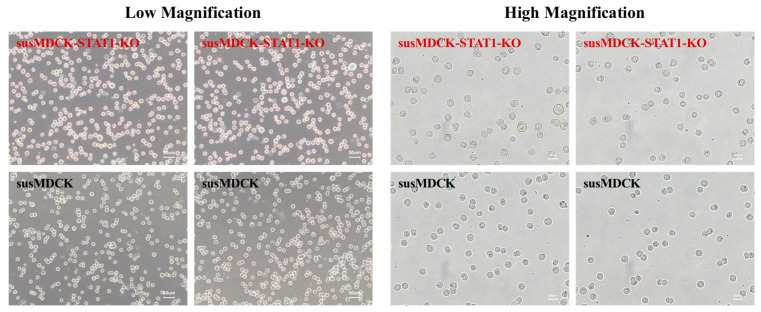
Morphological characteristics of cells under normal growth conditions.

Both the suspension-adapted susMDCK-STAT1-KO and susMDCK cells exhibited a smooth, spherical morphology, dispersing uniformly without significant aggregation, as shown in Figure 1A (see Figure A1 for more fields and lower magnification images). Following a synchronized infection with the H1N1 or H9N2 virus, both cell lines achieved comparable peak viable cell densities (~6 × 10^6^ cells/mL). However, their post-infection trajectories diverged markedly (Figure 1B). While both cell lines maintained similar viability under H1N1 infection, susMDCK-STAT1-KO cells displayed accelerated cell death upon H9N2 infection, commencing at 8 hpi. This accelerated cell death persisted, with an average specific death rate of 1.96 d^−1^ between 8–48 hpi. By 32 hpi, the viability in susMDCK-STAT1-KO cells had plummeted to 25%, significantly lower than susMDCK cells, which exhibited sustained activity and growth.

The cell diameter of both cell lines followed a similar trajectory during a synchronized infection with H1N1 and H9N2, initially increasing and peaking at 8 hpi before declining (Figure 1C). The maximum diameters of susMDCK-STAT1-KO cells under H1N1 and H9N2 infections were 17.70 μm and 17.67 μm, respectively, exceeding those of susMDCK cells by 0.69 μm and 1.15 μm. Throughout the infection period, susMDCK-STAT1-KO cells tended to exhibit larger diameters, suggesting an increased membrane surface area that may facilitate enhanced virus release.

During H1N1 infection, viral hemagglutinin (HA) titers in the supernatant were first detected at 3 hpi for susMDCK-STAT1-KO cells and at 4 hpi for susMDCK cells, with HA titers in susMDCK-STAT1-KO cells being consistently higher, suggesting a shorter viral replication cycle, as shown in Figure 1D. Maximum HA titers were reached at 16 hpi for susMDCK-STAT1-KO cells (11.33 log_2_HAU/100 μL) and at 24 hpi for susMDCK cells (10.72 log_2_HAU/100 μL). A similar trend was observed for H9N2 infection, with susMDCK-STAT1-KO cells producing higher overall HA titers, peaking at 13.06 log_2_HAU/100 μL compared to 11.55 log_2_HAU/100 μL for susMDCK cells. Notably, H9N2 HA titers were detectable as early as 3 hpi in both cell lines, with susMDCK-STAT1-KO cells showing higher titers than susMDCK cells. Regardless of the virus strain, infectious virus titers, as measured by TCID₅₀, were consistently higher in susMDCK-STAT1-KO cells (Figure 1E). The enhanced productivity of susMDCK-STAT1-KO cells was attributed to a greater single-cell virus yield (svy) and a higher virus release rate (Figure 1F). The maximum svy values for susMDCK-STAT1-KO cells were 9.01 × 10^3^ virions/cell for H1N1 and 28.34 × 10^3^ virions/cell for H9N2, representing 63% and 164% increases, respectively, compared to susMDCK cells.

These findings demonstrate that knocking out the STAT1 gene in susMDCK-STAT1-KO cells significantly enhances their capacity for influenza virus production, consistent with observations previously reported in adherent cell models [5]. Moreover, the serum-free suspension culture system adapted for susMDCK-STAT1-KO cells provides greater flexibility and scalability, making it a promising platform for large-scale viral production [17]. The replication of the influenza virus in cells is influenced by a complex interplay of factors, suggesting that STAT1 knockout may have broader effects on MDCK cells, impacting immune processes regulated by STAT1, the efficiency of the viral life cycle, and cellular metabolism.

### 3.2. Evaluation of Innate Immunity-Related Gene Expression in susMDCK-STAT1-KO Cells During Influenza Virus Infection

**Figure 2:**
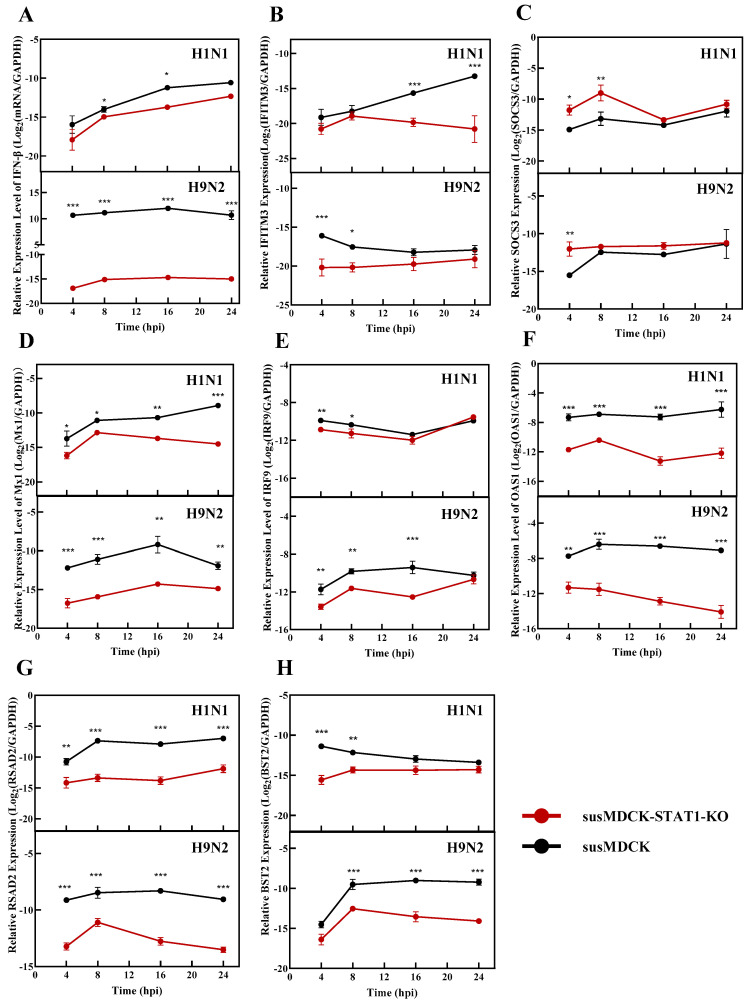
Transcriptional changes in immune-related genes in susMDCK-STAT1-KO and susMDCK cells post-synchronized IAV infection: (**A**) IFN-β; (**B**) IFITM3; (**C**) SOCS3; (**D**) Mx1; (**E**) IRF9; (**F**) OAS1; (**G**) RSAD2; and (**H**) BST2. All synchronized infections were performed with an MOI of 10, and gene expression levels were normalized to GAPDH, with * indicating 0.01 < *p* < 0.05, ** indicating 0.001 < *p* < 0.01, and *** indicating *p* < 0.001.

STAT1 plays a pivotal role in the cellular innate immune response [18]. To investigate the impacts of STAT1 knockout on innate immunity during viral replication, we examined the expression levels of key innate immunity-related genes in susMDCK-STAT1-KO cells, as shown in Figure 2. Notably, the transcription levels of Interferon beta (IFN-β) were significantly reduced in STAT1-deficient cells following viral infection, with the extent of reduction varying by virus type (Figure 2A). During H1N1 and H9N2 infections, the IFN-β transcription levels in susMDCK-STAT1-KO cells were approximately 10^−5^ relative to GAPDH, whereas, in susMDCK cells infected with H9N2, IFN-β levels exceeded a 1000-fold increase relative to GAPDH. This pronounced discrepancy likely reflects the higher entry and replication efficiency of the H9N2 virus.

Interferon-induced transmembrane protein 3 (IFITM3) and suppressor of cytokine signaling 3 (SOCS3), two crucial interferon-stimulated genes (ISGs) involved in viral adsorption and entry, exhibited differential expression patterns between the two cell lines. During both H1N1 and H9N2 infections, IFITM3 transcription was lower in susMDCK-STAT1-KO cells compared to susMDCK (Figure 2B), whereas SOCS3 transcription was higher (Figure 2C). IFITM3, a type II transmembrane protein, interacts with the influenza virus HA protein and disrupts lipid homeostasis, thereby inhibiting the fusion of the viral envelope with endosomal membranes and preventing the release of ribonucleoproteins (vRNP) into the cytoplasm [19]. In contrast, SOCS3 suppresses intracellular IFN signaling, thereby facilitating viral infection [20]. The reduced IFITM3 expression and increased SOCS3 expression in susMDCK-STAT1-KO cells may have contributed to enhanced viral entry efficiency.

The transcription levels of MX dynamin like GTPase 1 (Mx1), 2′-5′-oligoadenylate synthetase 1 (OAS1), and interferon regulatory factor 9 (IRF9), key ISGs involved in viral replication, were consistently lower in susMDCK-STAT1-KO cells compared to susMDCK cells during synchronized infection (Figure 2D–F). Mx1 inhibits viral mRNA and cRNA synthesis by obstructing the assembly of viral polymerase complexes [21], thereby suppressing the viral gene expression at both the transcriptional and translational levels. Similarly, OAS1 activates RNase to restrict viral genome replication, with its expression levels shown to be independent of the viral strain [22]. Our results align with these observations, as OAS1 transcription levels in susMDCK-STAT1-KO cells remained consistent across different viral infections. Additionally, IRF9 promotes ISG expression by forming the interferon-stimulated gene factor 3 (ISGF3) complex formation with STAT1 and STAT2 [23], thereby restricting viral replication. The reduced expression of these genes in susMDCK-STAT1-KO cells may facilitate more efficient viral RNA amplification.

Key ISGs involved in viral release, such as radical S-adenosyl methionine domain containing 2 (RSAD2) and bone marrow stromal cell antigen 2 (BST2), were also significantly downregulated in susMDCK-STAT1-KO cells during both H1N1 and H9N2 infections (Figure 2G,H). Viperin, encoded by the gene *RSAD2*, disrupts lipid raft formation and thus impedes influenza virus budding at these sites [24]. Similarly, tetherin, encoded by the gene *BST2*, anchors the enveloped viruses to the plasma membrane, preventing their release [25]. The reduced expression of these genes in susMDCK-STAT1-KO cells supports a potential mechanism underlying the enhanced efficiency of viral release.

Overall, alterations in the expression of various immune-related genes contribute to the efficient virus production in susMDCK-KO-STAT1 cells. The functions of these immune genes are intricately linked to viral entry, replication, and release, further emphasizing the pivotal role of STAT1 in innate immune responses.

### 3.3. Impact of STAT1 Knockout on Viral Entry, Gene Expression, Replication, and Release in susMDCK-STAT1-KO Cells

**Figure 3:**
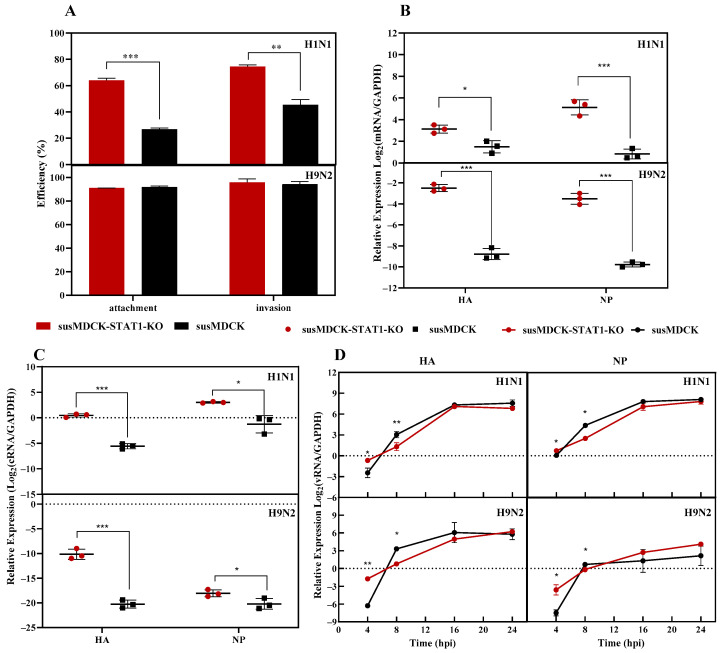
Differences in IAV infection cycle efficiency between susMDCK-STAT1-KO and susMDCK cells: (**A**) differences in the adsorption and uptake efficiencies of IAV; (**B**) intracellular levels of HA and NP mRNA at 4 hpi under synchronized infection; (**C**) intracellular levels of HA and NP cRNA at 4 hpi under synchronized infection; and (**D**) temporal changes in intracellular levels of HA and NP mRNA post-synchronized infection with IAV. Synchronized infections were performed with an MOI of 10, and gene expression levels were normalized to GAPDH, with * indicating 0.01 < *p* < 0.05, ** indicating 0.001 < *p* < 0.01, and *** indicating *p* < 0.001.

To determine the specific stages at which STAT1 knockdown affects influenza virus production in MDCK cells, we characterized the processes of viral entry, replication, and release in susMDCK-STAT1-KO cells, as shown in Figure 3. The results revealed that H1N1 adsorption efficiency was significantly higher in susMDCK-STAT1-KO cells (64%) compared to susMDCK cells (27%), while H9N2 adsorption efficiency exceeded 90% in both cell lines without significant differences (Figure 3A). Similarly, H1N1 invasion efficiency was markedly enhanced in susMDCK-STAT1-KO cells, whereas H9N2 invasion efficiency remained above 95% in both cell lines. These findings suggest that the increased H1N1 adsorption and invasion efficiency in susMDCK-STAT1-KO cells may accelerate the infection and replication processes, leading to earlier virus production. The lower adsorption and invasion efficiency of human-derived H1N1 compared to bird-derived H9N2 in MDCK cells is likely attributable to differences in the sialic acid receptor structures of MDCK cells [26].

During synchronized H1N1 infection, the transcription levels of HA and NP mRNA (Figure 3B) and cRNA (Figure 3C) at 4 hpi were significantly higher in susMDCK-STAT1-KO cells compared to susMDCK cells, with mRNA levels 1.95-fold and 25.35-fold higher, respectively. For H9N2, these differences were even more pronounced, with mRNA levels 129.70-fold and 89.51-fold higher. vRNA amplification kinetics showed a time-dependent increase in vRNA levels in both cell lines, stabilizing between 16 and 24 hpi (Figure 3D), consistent with patterns observed in adherent MDCK cells [27]. These findings underscore the critical role of viral gene transcription levels in determining the timing of virus release and production yield.

Interestingly, the vRNA levels of HA and NP genes displayed contrasting trends between 4 and 8 hpi. Specifically, vRNA levels were higher in susMDCK-STAT1-KO cells at 4 hpi; however, by 8 hpi, vRNA levels were significantly lower in susMDCK-STAT1-KO cells compared to susMDCK cells. Despite these differences, both cell lines exhibited similar vRNA levels during the stable phase between 16–24 hpi. This reversal may be attributed to the enhanced vRNA synthesis in susMDCK cells or the rapid release of vRNA-containing virions in susMDCK-STAT1-KO cells, leading to a depletion of intracellular vRNA levels.

To quantify this, the intracellular vRNA synthesis rate was approximated using NP vRNA copy numbers, under the assumption that each virion contains one NP vRNA. Intracellular NP vRNA copy numbers (~10^5^ copies/cell) were consistently two orders of magnitude higher than extracellular virus levels. However, as shown in Table 1, the vRNA release ratio in susMDCK-STAT1-KO cells was 2.8%, significantly exceeding the 0.86% observed in susMDCK cells—a 2.26-fold difference. These findings indicate that the superior influenza virus production capacity of susMDCK-STAT1-KO cells is primarily attributed to the enhanced viral release efficiency.

### 3.4. Effects of STAT1 Knockout on Membrane Synthesis and Related Amino Acid Utilization in susMDCK-STAT1-KO Cells During Influenza Virus Infection

**Figure 4:**
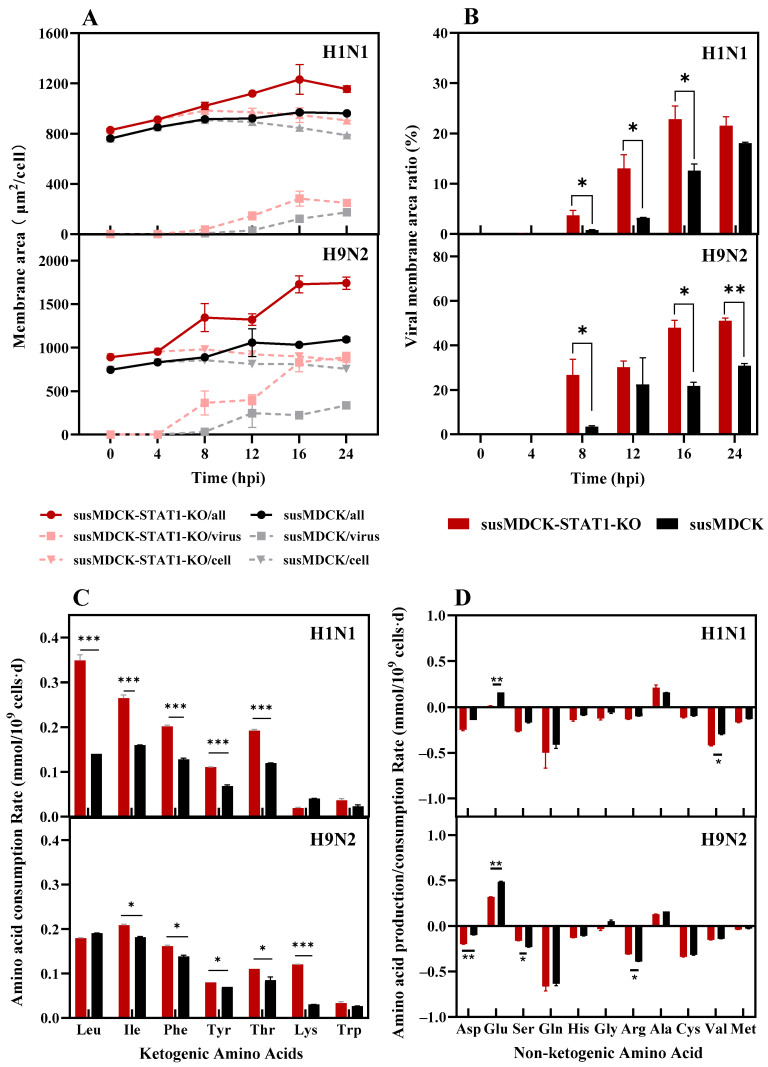
Differences in membrane synthesis and amino acid metabolism between susMDCK-STAT1-KO and susMDCK cells post-synchronized IAV infection: (**A**) changes in cell membrane area and viral membrane area; (**B**) proportion of viral membrane area over time; (**C**) differences in ketogenesis consumption rates from 0–24 hpi; and (**D**) differences in non-ketogenesis consumption rates from 0–24 hpi. Synchronized infections were performed with an MOI of 10, and * indicating 0.01 < *p* < 0.05, ** indicating 0.001 < *p* < 0.01, and *** indicating *p* < 0.001.

Enhanced viral replication and release in susMDCK-STAT1-KO cells confer a higher viral production capacity compared to susMDCK cells. The efficient viral replication observed may be linked to the downregulation of intracellular antiviral genes. However, the key factors influencing the viral release process remain unclear. To address this, we investigated changes in membrane synthesis and the related amino acid metabolism in susMDCK-STAT1-KO cells following influenza virus infection, as illustrated in Figure 4.

Assuming spherical shapes for both cells and viruses, with a virus particle diameter of 100 nm [28], membrane area changes were calculated based on cell diameters and single-cell virus yields, as shown in Figure 4A. During the first 16 hpi, the average total membrane synthesis rates in susMDCK-STAT1-KO cells were 25 μm^2^/cell/h and 53 μm^2^/cell/h under H1N1 and H9N2 infections, respectively, which were 1.94-fold and 2.90-fold higher than those in susMDCK cells (Table 2). During H1N1 infections, 10–20% of the synthesized membrane was utilized for virus budding, whereas, in H9N2 infections, over 50% of the membrane was used for virus release (Figure 4B). These results highlight the enhanced membrane synthesis capacity of susMDCK-STAT1-KO cells, which not only maintain membrane integrity but also provide sufficient membrane structures for the formation of viral envelopes, thereby facilitating viral release. However, the extensive depletion of membrane structures likely contributes to the rapid decline in cell viability and density after 16 hpi.

**Figure A2:**
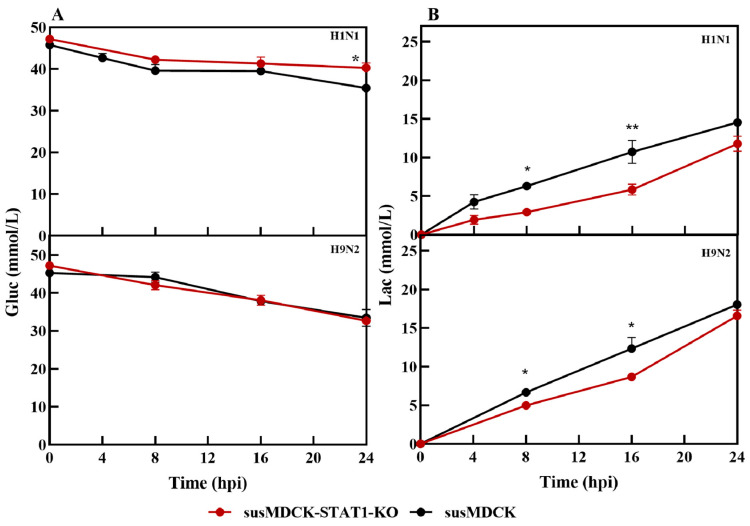
Changes in metabolites in the culture medium of susMDCK-STAT1-KO and susMDCK cells post-synchronized IAV infection: (**A**) glucose; and (**B**) lactate. Synchronized infections were performed with an MOI of 10, with * indicating 0.01 < *p* < 0.05, and ** indicating 0.001 < *p* < 0.01.

A metabolic analysis revealed no significant differences in glycolysis between the two cell lines (Table A3, Figure A2). However, notable differences were observed in the amino acid metabolism, particularly in the consumption of ketogenic amino acids (Figure 4C), such as leucine (Leu), isoleucine (Ile), phenylalanine (Phe), tyrosine (Tyr), and threonine (Thr). Among these, Leu and Ile were the most consumed ketogenic amino acids in both cell lines. These amino acids are metabolized into branched-chain α-keto acids in the cytoplasm, which are subsequently converted into acetyl-CoA in mitochondria [29]. Acetyl-CoA serves as a critical precursor for fatty acid and sterol biosynthesis, providing raw materials for membrane formation [30]. For instance, during H1N1 infection, susMDCK-STAT1-KO cells exhibited a Leu-specific consumption rate of 0.35 mmol/10^9^ cells·d, which was 2.5-fold higher than that of susMDCK cells under the same conditions (Figure 4C). Similarly, the consumption rates of Phe, Tyr, and Thr in susMDCK-STAT1-KO cells were 1.23–1.72 times higher than those in susMDCK cells, highlighting an enhanced capacity for acetyl-CoA production to support lipid biosynthesis. Non-ketogenic amino acid metabolism exhibited less uniformity (Figure 4D). Glutamine consumption rates ranged between 0.4–0.7 mmol/10^9^ cells·d for both cell lines, with no significant differences; however, glutamate production rates were significantly lower in susMDCK-STAT1-KO cells during both infections. The consumption patterns of other non-ketogenic amino acids varied depending on the viral strain, suggesting strain-specific differences in amino acid requirements, which warrant further investigation.

These findings indicate that the superior membrane synthesis capacity of susMDCK-STAT1-KO cells is supported by the elevated consumption of ketogenic amino acids, facilitating the maintenance of cellular integrity and robust virus release. However, the strain-specific differences in amino acid utilization underscore the complexity of the metabolic adaptations required for efficient influenza virus production. Further mechanistic studies are needed to elucidate the precise metabolic pathways underlying these observations.

### 3.5. The Role of Cholesterol for Enhanced Viral Production in susMDCK-STAT1-KO Cells

**Figure 5:**
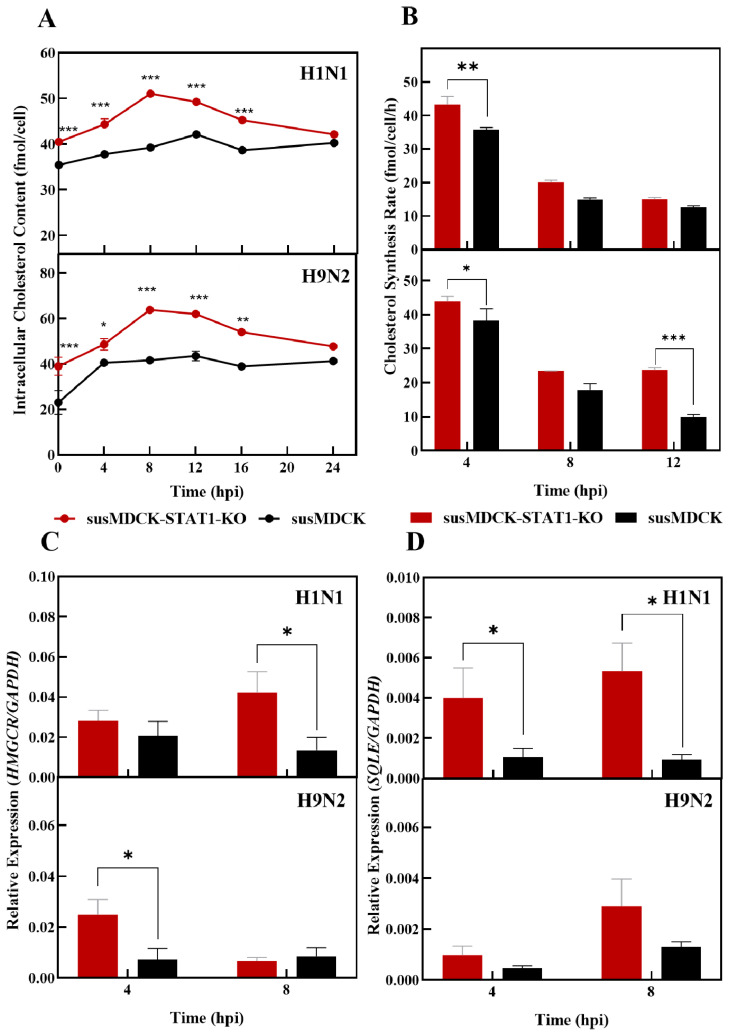
Differences in cholesterol metabolism between susMDCK-STAT1-KO and susMDCK cells post-synchronized IAV infection: (**A**) changes in intracellular cholesterol content; (**B**) variations in cholesterol synthesis rates; (**C**) differences in HMGCR transcription levels; and (**D**) differences in SQLE transcription levels. Synchronized infections were performed with an MOI of 10, and gene expression levels were normalized to GAPDH, with * indicating 0.01 < *p* < 0.05, ** indicating 0.001 < *p* < 0.01, and *** indicating *p* < 0.001.

A rapid and sustained virus release depletes cellular membranes, whose regeneration depends heavily on host lipid metabolism [31]. Given the pivotal role of cholesterol in cellular membranes (approximately 40%) [32] and influenza virions (11–12%) [33], we sought to investigate its impact on influenza virus synthesis in susMDCK-STAT1-KO cells. Specifically, we examined alterations in intracellular cholesterol levels and the differential expression of rate-limiting enzymes in both susMDCK-STAT1-KO and susMDCK cells following synchronous infection, as illustrated in Figure 5.

susMDCK-STAT1-KO cells exhibit significantly elevated intracellular cholesterol levels compared to susMDCK cells, irrespective of the infecting virus (Figure 5A). At 8 h post-infection (hpi), the intracellular cholesterol content in susMDCK-STAT1-KO cells reached 51.04 fmol/cell during H1N1 infection and 63.75 fmol/cell during H9N2 infection, corresponding to 1.37- and 1.54-fold increases, respectively, compared to the peak levels observed in susMDCK cells. Cholesterol synthesis rates at the single-cell level were highest during the 0–4 hpi period for both cell lines, ranging from 30 to 45 fmol/cell/h, before declining to below 25 fmol/cell/h (Figure 5B). The synthesis rate of cholesterol in susMDCK-STAT1-KO cells was consistently higher than in susMDCK cells, with the maximum observed difference reaching 1.7-fold, suggesting a more robust cholesterol metabolism in STAT1-deficient cells.

Cholesterol facilitates membrane fusion during viral entry, promoting the release of the virus [34]. During viral budding, influenza viruses incorporate cholesterol-enriched host membranes into their envelopes, making the cholesterol content and synthesis capacity of host cells crucial to virus production. In susMDCK-STAT1-KO cells, the enhanced cholesterol metabolism supports the formation of lipid rafts, which facilitate viral release, while simultaneously providing structural material for both the cellular membranes and viral envelopes. This increased metabolic capacity likely underpins the cells’ ability to sustain efficient viral release despite the heightened membrane demands, thereby contributing to increased viral production and titers. Our findings indicate that influenza virus infection stimulates intracellular cholesterol synthesis, a phenomenon also observed in other enveloped viruses, such as gamma herpesvirus, human cytomegalovirus, and classical swine fever virus [35,36,37], suggesting that elevated cholesterol synthesis may be a common feature of enveloped virus infections.

Cholesterol biosynthesis involves over 20 enzymatic reactions, with HMG-CoA reductase (HMGCR) and squalene monooxygenase (SQLE) identified as key rate-limiting enzymes [38]. Between 4–8 hpi, transcriptional levels of HMGCR and SQLE were consistently elevated in susMDCK-STAT1-KO cells compared to susMDCK cells, particularly at 8 hpi during H1N1 infection (Figure 5C,D). This upregulation likely contributes to the increased cholesterol synthesis observed in STAT1-deficient cells.

Furthermore, innate immune signaling plays a critical role in regulating cholesterol transport, storage, and secretion [39,40]. Interferon (IFN) inhibits cholesterol synthesis by downregulating the expression of sterol regulatory element-binding protein 2 (SREBP2), thus suppressing viral replication [41]. In susMDCK-STAT1-KO cells, reduced IFN transcription levels and attenuated innate immune responses may relieve the IFN-mediated suppression of SREBP2, thereby further promoting intracellular cholesterol synthesis. More importantly, STAT1 directly influences cellular lipid metabolism. On the one hand, STAT1 induces the expression of cholesterol 25-hydroxylase (Ch25h), stimulating the synthesis of the antiviral compound 25-hydroxycholesterol (25HC) [42]. The knockout of STAT1 impairs this process. Additionally, STAT1 knockout reduces the expression of 1-acyl-CoA synthetase long-chain family member 1 (ACSL1), leading to decreased lipid oxidation levels [43]. This reduction in lipid oxidation likely contributes to the reduced membrane damage in susMDCK-STAT1-KO cells, potentially facilitating viral release.

### 3.6. Critical Role of Cholesterol in Viral Production: Insights from Cholesterol Deprivation

**Figure 6:**
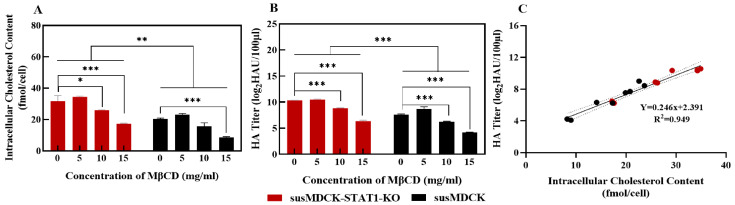
Impact of cholesterol depletion on IAV production in susMDCK-STAT1-KO and susMDCK cells: (**A**) effect of MβCD on intracellular cholesterol content; (**B**) effect of MβCD on the HA titer of produced viruses; and (**C**) correlation between cholesterol content and HA titers. All infections and virus production were conducted under synchronized infection conditions, with an MOI of 10, with * indicating 0.01 < *p* < 0.05, ** indicating 0.001 < *p* < 0.01, and *** indicating *p* < 0.001.

To further elucidate the critical role of cholesterol in viral production, we performed a cholesterol deprivation assay, as depicted in Figure 6. Methyl-β-cyclodextrin (MβCD), which exhibits a high affinity for cholesterol, forms soluble inclusion complexes that effectively extract cholesterol from cells. Increasing concentrations of MβCD resulted in a progressive reduction in intracellular cholesterol content (Figure 6A), which was accompanied by a concomitant decrease in viral hemagglutinin (HA) titers (Figure 6B). At an MβCD concentration of 15 mg/mL, intracellular cholesterol levels in susMDCK-STAT1-KO and susMDCK cells were reduced to 17 fmol/cell and 8.61 fmol/cell, respectively, reflecting reductions of 45.18% and 57.62% compared to untreated controls. In parallel, HA titers decreased to 6.35 log_2_HAU/100 µL and 4.16 log_2_HAU/100 µL, representing declines of 39.50% and 45.41%, respectively. Notably, at the same MβCD concentration, susMDCK-STAT1-KO cells retained higher intracellular cholesterol levels and HA titers compared to susMDCK cells.

A linear regression analysis of intracellular cholesterol content and HA titers at 6 hpi under various MβCD treatments revealed a robust positive correlation (R^2^ = 0.949), indicating that higher intracellular cholesterol levels within a defined range are associated with increased viral production (Figure 6C). Additionally, statins, which inhibit enzymes involved in intracellular cholesterol biosynthesis [44], have been shown to significantly reduce intracellular viral vRNA levels and HA titers in MDCK cells treated with simvastatin [45]. These findings suggest that both exogenous cholesterol depletion via MβCD and the endogenous inhibition of cholesterol biosynthesis by statins impair viral production, highlighting the essential role of intracellular cholesterol in this process.

**Figure 7:**
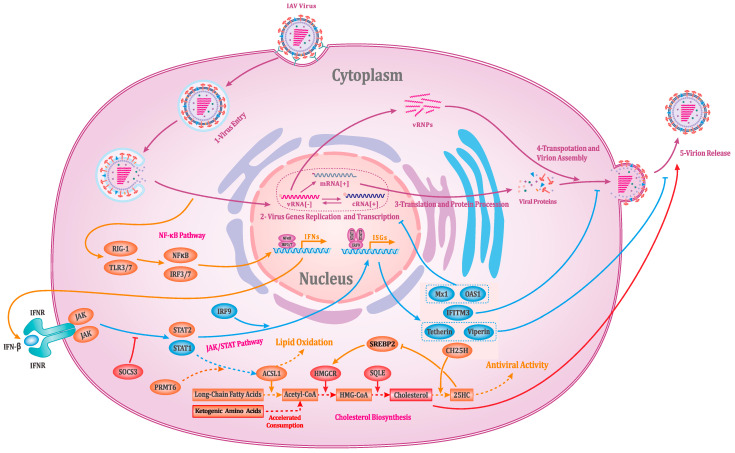
Summary of the differential expression of immune-related genes and metabolites in susMDCK-STAT1-KO cells and their impact on viral replication. In the figure, gene expression products are represented by circles, except for IFNR, which is depicted using an irregular shape, and metabolites are represented by squares. The names of these elements are labeled within or near their respective shapes. Genes or metabolites marked in red are significantly upregulated in susMDCK-STAT1-KO cells, while genes marked in blue are significantly downregulated. Elements marked in orange indicate potential associations with differentially expressed genes or metabolites. The effects of these differentially expressed genes or metabolites on the pathways and influenza virus replication are highlighted using the corresponding differential expression colors.

Overall, the knockout of the STAT1 gene in susMDCK-STAT1-KO cells has a wide-ranging impact on the intracellular environment following influenza virus infection, as shown in Figure 7. On one hand, the JAK/STAT signaling pathway, directly associated with STAT1, is suppressed to varying degrees at different stages, leading to a significant downregulation of multiple ISGs activated by this pathway. For instance, the reduced expression of Mx1 and OAS1 alleviates their inhibitory effects on the replication and expression of the influenza virus genome, while the downregulation of IFITM3, Tetherin, and Viperin facilitates the assembly and release of the influenza virus. On the other hand, the knockout of STAT1 appears to influence lipid metabolism processes. Specifically, susMDCK-STAT1-KO cells exhibit a faster membrane synthesis rate and higher intracellular cholesterol levels, accompanied by an increased consumption rate of related metabolic substrates such as ketogenic amino acids and elevated transcription levels of key genes such as HMGCR and SQLE. Additionally, experiments using MβCD to deprive intracellular cholesterol further underscore the critical role of cholesterol in supporting influenza virus production. Although no studies have directly reported the relationship between STAT1 knockout and lipid metabolism during influenza virus infection, innate immune signaling has been shown to play a critical role in regulating cholesterol metabolism [39,40,41,42,43]. In susMDCK-STAT1-KO cells, STAT1 knockout likely reduces the expression of Ch25h, which may relieve its suppression of SREBP2, thereby promoting cholesterol synthesis [46]. Moreover, the knockout of STAT1 in susMDCK-STAT1-KO cells may have reduced the expression level of ACSL1. This reduction not only impacts lipid metabolism but also contributes to a decrease in lipid oxidation levels [43], thereby alleviating plasma membrane damage and potentially facilitating the release of budding-type viruses. However, to precisely elucidate the molecular processes influenced by cholesterol and to leverage them for cell line development or process optimization, a broader characterization of gene expression across intracellular pathways will be required.

## 4. Conclusions

In conclusion, this study confirms the high efficiency of IAV production in suspension-adapted susMDCK-KO-STAT1 cells, with single-cell virus yields for human-derived H1N1 and avian-derived H9N2 strains exceeding those of suspension-adapted susMDCK cells by 63% and 164%, respectively. In addition to boosting IAV replication through modulation of various innate immunity-related genes, this study further reveals that the STAT1 knockout significantly enhances the cells’ capacity for membrane and cholesterol synthesis. The cholesterol deprivation assays also highlight the crucial role of intracellular cholesterol in IAV production, demonstrating that its depletion markedly reduces viral titers. These findings not only deepen our understanding of cell-based influenza virus production in serum-free suspension culture systems but also provide valuable insights for the development of a higher-yield, more efficient vaccine production process. With the approval of several MDCK cell-based influenza vaccines and the establishment of multiple cell-based vaccine production platforms, the study lays the groundwork for future strategies, such as the concurrent modification of the JAK/STAT pathway and lipid metabolism-related pathways, to enhance viral replication and optimize viral release synergistically.

## Figures and Tables

**Table 1:** Calculation of intra- and extracellular virion releasing ratio at 16 hpi synchronously infected with H1N1.

	susMDCK-STAT1-KO	susMDCK
Intracellular vRNA Copies	(3.44 ± 0.86) × 10^5^	(4.54 ± 0.50) × 10^5^
Released Virion Particles	(9.01 ± 1.33) × 10^3^	(3.89 ± 0.33) × 10^3^
Release Ratio (%)	2.8 ± 1.05	0.86 ± 0.16

Note: The unit for the intracellular vRNA copy rate is expressed as copies per cell, whereas the unit for released virion particles is expressed as virions per cell.

**Table 2:** Membrane synthesis rate from 0–16 hpi, and cell dry weight and lipid dry weight at 8 hpi hpi in synchronous infection.

	H1N1		H9N2	
	susMDCK-STAT1-KO	susMDCK	susMDCK-STAT1-KO	susMDCK
Membrane Synthesis Rate	25.23 ± 6.65	13.01 ± 1.58	52.83 ± 6.83	18.05 ± 1.03
Cell Dry Weight	42.0 ± 3.0	36.25 ± 2.75	38.75 ± 0.75	31.5 ± 0.1
Lipid Dry Weight	4.7 ± 0.1	3.85 ± 0.05	6.15 ± 0.15	4.25 ± 0.05
Ratio (%)	11.26 ± 1.04	10.69 ± 1.9	15.88 ± 0.60	13.51 ± 0.58

Note: The unit for membrane synthesis rate is μm^2^/cell/h, while the units for cell dry weight and lipid dry weight are mg/10^8^ cells.

## Data Availability

Additional data are available upon request from the corresponding author.

## Appendix A

**Table A1:** Primer sequences for reverse transcription.

Primer Name	Sequence
H1N1HA-cRNA	GCTAGCTTCAGCTAGGCATCAAACAAGGGTGTTTTTCCTCATATCTCTGAA
H1N1-HA-mRNA	CCAGATCGTTCGAGTCGTTTTTTTTTTTCCTCATATCTCTGAAAT
H1N1-HA-vRNA	GGCCGTCATGGTGGCGAATGAGATTTACCCCGGAAATAGCAGA
H1N1-NP-cRNA	GCTAGCTTCAGCTAGGCATCAACAAGGGTATTTTTCTTTAATTGTCGTAC
H1N1-NP-mRNA	CCAGATCGTTCGAGTCGTTTTTTTTTTTCTTTAATTGTCGTACTCC
H1N1-NP-vRNA	GGCCGTCATGGTGGCGAATGAACAATGGTGATGGAATTGGTCA
H9N2-HA-cRNA	GCTAGCTTCAGCTAGGCATCAGTAGAAACAAGGGTGTTTTTGCTAATTATATAC
H9N2-HA-mRNA	CCAGATCGTTCGAGTCGTTTTTTTTTTTTTTTTTGCTAATTATATACAAAT
H9N2-HA-vRNA	GGCCGTCATGGTGGCGAATGGGTCAAACACTGCGAATAAAATCTAATGG
H9N2-NP-cRNA	GCTAGCTTCAGCTAGGCATCAGTAGAAACAAGGGTATTTTTCTTCAATTGTC
H9N2-NP-mRNA	CCAGATCGTTCGAGTCGTTTTTTTTTTTTTTTTTCTTCAATTGTCATACTC
H9N2-NP-vRNA	GGCCGTCATGGTGGCGAATGATCAAGTGCGAGAGAGCAGAAAT

**Table A2:** Primer sequences for qRT-PCR.

Primer Name	Sequence
Tag-cRNA	GCTAGCTTCAGCTAGGCATC
Tag-mRNA	CCAGATCGTTCGAGTCGT
Tag-vRNA	GGCCGTCATGGTGGCGAAT
H1N1-HA-cRNA	GGAGTGAAATTGGAATCAATGGGG
H1N1-HA-mRNA	GGAGTGAAATTGGAATCAATGGGG
H1N1-HA-vRNA	ACCCAAAGCCTCTACTCAGTGCG
H1N1-NP-cRNA	AGAACATCTGACATGAGGACCG
H1N1-NP-mRNA	AGAACATCTGACATGAGGACCG
H1N1-NP-vRNA	CCTGGGTTCCGGCTCTCTCTCA
H9N2-HA-cRNA	GGTCAAGCTGGAATCTGAAGGAACTTAC
H9N2-HA-mRNA	GGTCAAGCTGGAATCTGAAGGAACTTAC
H9N2-HA-vRNA	GTGTTTAAGCCACCCTTCTCTGTCTG
H9N2-NP-cRNA	CAGAAGATGTGTCATTCCAGGGGC
H9N2-NP-mRNA	CAGAAGATGTGTCATTCCAGGGGC
H9N2-NP-vRNA	CATATCCACTGGCCACTGCAAGTC
GAPDH-F	GTAGTGAAGCAGGCATCGGA
GAPDH-R	GTCGAAGGTGGAAGAGTGGG
IFN-β-F	AGCAGCAGTTTGGAGTGTCA
IFN-β-R	CTCCTTCTGGAACTGGCGTG
IFITM3-F	ACCAAGACCAGTACAAGGTTCC
IFITM3-R	GATGTGGTCGGGGCAC
SOCS3-F	TGTCGGAAGACGGTCAATGG
SOCS3-R	CCCCTGCCCTTTGCTCTTTA
Mx1-F	ATTCAGATTGGGGGTGGACG
Mx1-R	ATGCTTTGCATGCACTGGTC
OAS1-F	ACACAGGCATGACACAGTCC
OAS1-R	AACCCTCCCGAACACCAAAC
IRF9-F	TCAAGGCGTGGGCCATATTT
IRF9-R	GTCCCTATGGCCGTTCTCAG
RSAD2-F	GAAGATGGCTGAGACCACCC
RSAD2-R	AGAGCCTCATGGAGACTGGT
BST2-F	TCACAATGTCACCAGCCTCC
BST2-R	CAGGCCTTCTCCTTCACCAG
HMGCR-F	AGACCTCCAAGGAACTTGCG
HMGCR-R	TCCTCAACACAACACCGCAT
SQLE-F	AGACGCCGGCGATTCCG
SQLE-R	GTAGGGTTCTGGCGATCCG

**Table A3:** Glucose and lactate metabolism of susMDCK-STAT1-KO and susMDCK cells in synchronous infection.

	H1N1		H9N2	
	susMDCK-STAT1-KO	susMDCK	susMDCK-STAT1-KO	susMDCK
q_Gluc_	−1.32 ± 0.18	−1.30 ± 0.14	−1.73 ± 0.06	−1.89 ± 0.06
q_Lac_	1.68 ± 0.29	1.58 ± 0.15	2.16 ± 0.11	2.01 ± 0.16
Y_Lac/Gluc_	1.33 ± 0.40	1.22 ± 0.07	1.25 ± 0.11	1.06 ± 0.11
